# Predicting Statistical Properties of Open Reading Frames in Bacterial Genomes

**DOI:** 10.1371/journal.pone.0045103

**Published:** 2012-09-24

**Authors:** Katharina Mir, Klaus Neuhaus, Siegfried Scherer, Martin Bossert, Steffen Schober

**Affiliations:** 1 Institute of Communications Engineering, Ulm University, Ulm, Germany; 2 Chair for Microbial Ecology, Technische Universität München, Freising, Germany; Louisiana State University and A & M College, United States of America

## Abstract

An analytical model based on the statistical properties of Open Reading Frames (ORFs) of eubacterial genomes such as codon composition and sequence length of all reading frames was developed. This new model predicts the average length, maximum length as well as the length distribution of the ORFs of 70 species with GC contents varying between 21% and 74%. Furthermore, the number of annotated genes is predicted with high accordance. However, the ORF length distribution in the five alternative reading frames shows interesting deviations from the predicted distribution. In particular, long ORFs appear more often than expected statistically. The unexpected depletion of stop codons in these alternative open reading frames cannot completely be explained by a biased codon usage in the +1 frame. While it is unknown if the stop codon depletion has a biological function, it could be due to a protein coding capacity of alternative ORFs exerting a selection pressure which prevents the fixation of stop codon mutations. The comparison of the analytical model with bacterial genomes, therefore, leads to a hypothesis suggesting novel gene candidates which can now be investigated in subsequent wet lab experiments.

## Introduction

**Figure 1:**
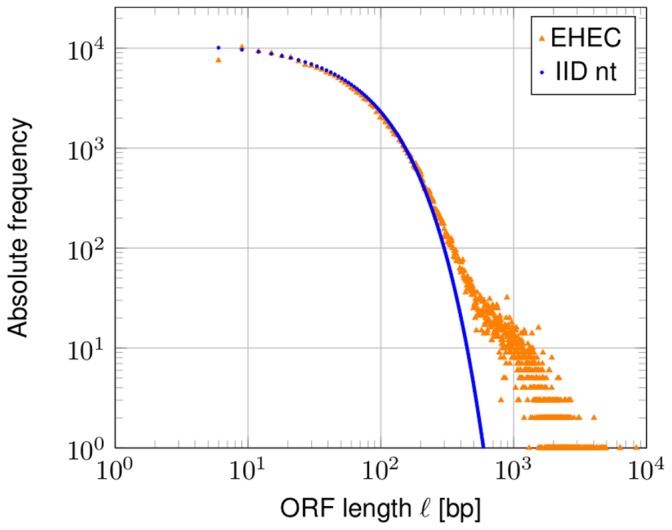
ORF lengths distribution of *Escherichia coli* O157:H7 Sakai. ORF lengths are given in base pairs (bp). All ORFs in the six possible reading frames are shown. The prediction of a simple model based on independent and identically chosen nucleotides (IID nt) is not able to reproduce the ORF distribution.

The physical basis for heredity is the DNA double helix. Proteins are encoded in Open Reading Frames (ORFs) delimited by a start and stop codon. In prokaryotes, genes act as a basic organizational unit at the genome level, since the coding density of bacterial genomes is quite high compared to eukaryotes [1]. The genome of a typical bacterium is somewhere in the range of 10^6^ to 10^7^ base pairs (bp), containing about 10^3^ to 10^4^ annotated genes. However, the total number of possible ORFs is usually in the order of 10^4^ to 10^5^. Although the number and the typical length of ORFs may vary, bacteria share common characteristics of their open reading frame length distribution, which is correlated to their GC-content. Most ORFs are rather short. Investigating the statistical properties of a genome with GC-content 21.4%, we observe that 75% of all ORFs are shorter than 15 codons. On the other hand, only 0.1% of all ORFs have lengths larger than 779 codons. The same tendency holds for a genome with a high GC-content of 75.9%, in this case 75% of all ORFs are shorter than 195 codons and a minority of 0.1% are larger than 1854 codons (own data). It is a well-known fact that the distribution of the overall ORF lengths correlates with the GC-content of a genome, simply because stop codons being AT-rich. The GC-content of a genome also governs overall codon usage in a genome [2], [3]. Oliver *et al.* [2] calculated a theoretical stop codon probability depending on the GC-content, and the expected distribution of ORF lengths in a random model of independent and identically (IID) chosen nucleotides. They found for the latter that the probability to observe an ORF comprising more than 200 codons is rather small, despite varying the GC-content from 30% to 70% However, their considerations are overly simplistic, since the genetic code does not allow an IID behavior of the nucleotides (Figure 1, IID nt). Since most parts of bacterial genomes are covered by genes, the general statistical behavior of bacterial genomes is expected to be determined by the codon usage.

Distributions of ORF lengths in bacterial genomes have been studied by Li [4] in some detail. An example of such a length distribution is shown in Figure 1 for the genome of *Escherichia coli* O157:H7 Sakai (EHEC, accession NC_002695).

Li [4] proposed a piece-wise exponential model for bacterial ORF length distributions. The length *L* of a single ORF is described using the probability density function
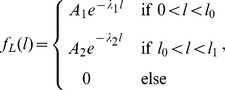
for rates 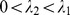 and positive normalizing constants *A*_1_ and *A*_2_. In the region of shorter ORFs (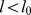), the exponential distribution decays faster, whereas the region of longer ORFs depicts a slower decay rate. The parameter denotes the transition point between the two exponential functions and parameter *l*_1_ is determined by the maximal ORF length of a given genome. The length distributions of four archaeal, 13 eubacterial, and one eukaryotic genome have been studied by this author. Interestingly, the author concluded that *l*_0_ is ∼400 base pairs, irrespective for the organism studied. However, the equation proposed by Li [4] is empirical only, and gives no mathematical explanation why such a transition point *l*_0_ exists.

In a more recent study, McCoy *et al.* [5] proposed a different model describing the natural ORF lengths as a mixture model of two distributions
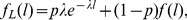
(1)for rate 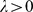, where *p* denotes a weighting factor and 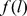 represents the density function of either a lognormal or a gamma distribution, depending on the organism investigated. The decision which distribution is chosen was based on Akaike’s Information Criterion. The authors noted that the exponential part of Equation (1) is connected to random IID models of nucleotides. Their choice of 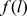 was motivated by the fact that the length distribution of the annotated proteins is empirically described either by a lognormal or a gamma distribution [6] with only minor differences in 297 completely sequenced bacterial and 14 eukaryotic genomes. For short ORFs, McCoy *et al.* [5] predict a one-to-one correspondence between 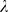, which is estimated based on the observed size distribution of ORFs and the stop codon probability [2]. This is due to the fact that the length of random sequences between successive occurrences of stop codons follows a geometric distribution [7], which is approximated by an exponential distribution in [5]. Note that the same holds for the length distribution between a start and a stop codon. According to Oliver *et al.* [2] the parameter of the exponential distribution is the probability that a nucleotide triplet is a stop codon.

In this work, a random IID sequence, called *Rcodon*, based solely on the codon usage of the bacterial genome and the genome length is investigated. Additionally, we derived a theoretical model, called *mixture model*, which is an approximation of the artificial genome *Rcodon*, using average case analyses and stationarity assumptions (see *Materials and Methods*). We used *Rcodon* to verify the predictions of our analytical mixture model. Several global properties of the underlying bacterial genome, such as the total number of ORFs, the ratio of coding to non-coding ORFs and the global ORF length distribution itself are predicted by the model. We calculate the maximal ORF length that can be derived from the model for each individual reading frame. Furthermore, the influence of the GC-content and the sequence length on the number of ORFs and the average ORF length directly follows from the model. Thus, many over-all aspects of bacterial genomes are attributable to codon usage statistics. Since our model is based on statistical and not on empirical properties, deviations between the model and bacterial genomes are a powerful predictor of evolutionary constraints, which is in contrast to former proposals.

The interest of this paper lies in the statistical properties of ORFs in order to investigate the potential existence of overlapping genes. This term refers to a DNA locus encoding two proteins in two different reading frames. Arrangements of such overlapping genes have long been acknowledged in viruses. For instance, the first completely sequenced genome, bacteriophage Φ*X*174, displays a number of such overlapping genes [8]. However, viruses are thought to be special cases due to genome size restrictions caused by space limitations of the capsid volume [9]. For the most part, overlapping ORFs in alternative reading frames are omitted in bacterial genome annotations [10]–[12] due to obvious information content constraints [13]. Since bacterial genomes are non-random strings of nucleotides, we hypothesize that – if protein coding genes exist in alternative, overlapping reading frames – the statistical parameters in overlapping frames should be different from random expectation. Already in 1994, Merino *et al.* [14], suggested that long ORFs in antisense (thus, alternative reading frames −1 to −3) are a frequent, non-random phenomenon in all organisms, primarily caused by codon usage. They also hypothesized that especially the long ORFs in frame −1 could relate to an ancient genetic translation system preferring certain codons [14]. However, most authors tended to reject overlapping genes due to a so called “information content constrains” as the major argument in later years (e.g., [10], [11], [13]). This constraint should limit evolution, since two genes are interlocked. Despite, several overlapping genes have been described in recent years, both from eukaryotes [15] and bacteria, to which our study is limited. Jensen *et al.* [16] re-annotated the genome of a *Chlamydia* species using a new gene-finder program. Fifteen novel genes have been predicted overlapping to already annotated genes. Transcription and other circumventive evidence let the authors conclude that at least seven of those are protein coding. The overlapping gene pair *htgA/yaaW* from *E. coli* was considered to be overlapping, but *htgA* has been removed from the annotated genome due to conflicting data [17], [18]. However, a plasmid-encoded gene from *E. coli*, *tnpA*, forms a transposase-like protein and contains *astA*, a heat stable enterotoxin [19], [20]. In a close relative to *E. coli*, *Shigella flexneri*, the overlapping gene pair *pic/setAB* had been identified. *pic* encodes a mucinase which digests intestinal mucus and the genes *setAB* an enterotoxin [21]. A series of publications about *Pseudomonas fluorescens* identified several overlapping genes in this organism (e.g., [22], [23]). For some of the overlapping genes the protein-products have been identified using mass spectrometry [24]. Last but not least, Tunca *et al.* [25] used nicely designed strand specific knock-out mutants to demonstrate a phenotype for both genes of the overlapping gene pair *dmdR1/adm*. The former is a homolog of a well-known class of iron regulators, the latter turned out to be involved in the control of secondary metabolites. Both genes overlap except a few base pairs in antiparallel fashion and are about 700 bp in length [25]. Thus, increasing evidence as cited above, from many different unrelated bacteria suggests that overlapping genes are no rare biological oddities. In this work, we show that the amount of long ORFs in alternative frames of bacterial genomes exceeds theoretical expectations.

## Results and Discussion

### A Random Model Genome

Due to its triplet code, double stranded DNA can encode six different reading frames. Three frames [+1, +2, +3]on the DNA strand in 5′ to 3′ direction and further three frames on the antisense strand [−1, −2, −3]. Throughout this work, reading frame +1 is defined as the frame in which an annotated gene is located. An open reading frame is defined as the region between a start codon *NTG*, with 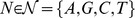, followed by number of triplets (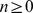) and concluded with one of the three possible stop codons [*TAG,TGA,TAA*]. Although some of the start codons are rare (e.g., *TTG,CTG* [26]–[28]) they are used in bacteria and are only a single point mutation away from the preferred start codons *ATG* or*GTG* [29], [30]. The total length of an ORF is given in base pairs (bp) including the start and stop codon. We also use a measure counting the codons within an ORF ignoring the stop codon, since the latter is not translated into an amino acid (AA).

In this paper, most results presented are exemplified using the genome of the pathogenic *Escherichia coli* O157:H7 Sakai (EHEC, accession NC_002695, [27]), which is an important zoonotic and food borne organism [31]–[34]. The GC-content is ∼50%. The artificial random genome model *Rcodon* is derived from the codon usage of the respective natural genome. It comprises a random sequence of IID codons of the organism investigated which has the same length as the bacterial genome. All ORFs in frame+1 are considered as “annotated” ORFs of *Rcodon* by definition. Further bacterial genomes examined with other GC-contents are listed in Table 3. Results for those organisms are mentioned if appropriate.

**Figure 2:**
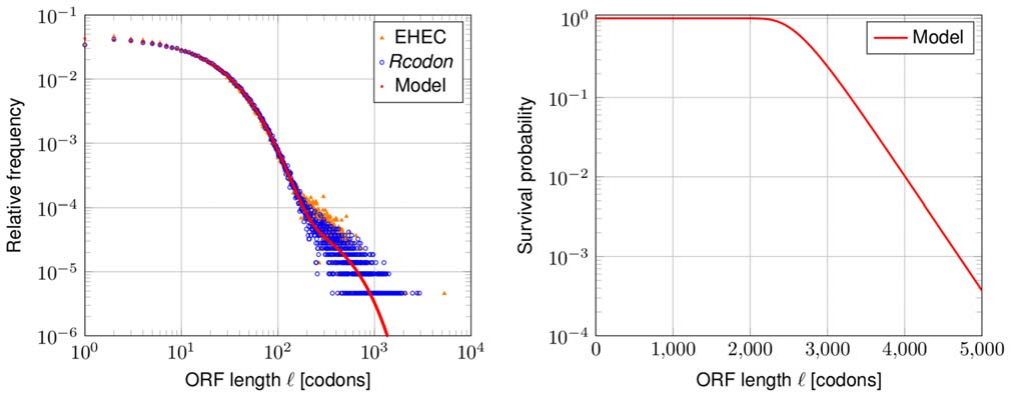
ORF lengths distribution and survival probability. Left panel: Shown is the relative frequency of the EHEC ORF lengths (orange triangles) and of *Rcodon* (blue open dots). The prediction of the mixture model is shown in red. Right panel: Survival probability (probability to observe at least one ORF with given length 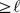 in any of the six reading frames) of the mixture model.

The global length distribution of the EHEC genome compared to its artificial *Rcodon* genome shows excellent correspondence (Figure 2, left panel). The same overall features of a high number of short ORFs and lesser numbers of longer ORFs is observable despite the fact that *Rcodon* depends on a relatively simple concept. At this stage it is unclear, however, whether long ORFs are a consequence of selective forces or whether they do appear randomly. It would be helpful to distinguish between ORFs that either can be traced to evolutionary selection or to simple statistical properties of the considered genome.

### Derivation of a Predictive Theoretical Model

The random process of drawing codons until the first stop codon appears follows a geometric distribution. Thus, the ORF lengths in frame +1 are intuitively described by such a distribution and depend on the stop codon probability only. However, certain codons in frame +1 cause stop codons in other frames. For instance, the leucine codon *CAT* causes a stop codon *TAG* in frame −1. Similarly, combinations of certain pairs of codons in +1 cause stop codons in other alternative frames. Therefore, the length distribution of the ORFs in each frame depends on the codon usage of frame +1 and each distribution in an alternative frame follows a different geometric distribution, depending on the probability of codons or pairs of codons in +1 forming a stop codon in the respective alternative frame. Thus, we developed a mixture model of six geometric distributions, which closely follows the natural distribution (Figure 2, left panel).

A detailed derivation of the model can be found in the *Materials and Methods* part. The probability to observe exactly one ORF of length 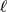 in any of the six reading frames is calculated according to Equation (3) by
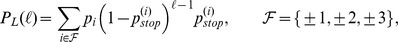
where 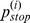 denotes the stop codon probability in an individual reading frame *i* and 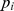 is a weighting factor; 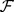 denotes 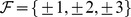 in short. The weighting factor is calculated according to Equation (6) by



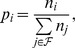
in conjunction with Equation (5)



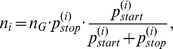
where 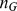 is the sequence length and 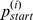 denotes the start codon probability in an individual reading frame 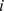. The probability to observe at least one ORF with minimum length 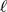 in 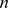 trials, where 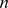 is the number of ORFs calculated via 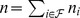, is called the *survival probability*, which is calculated following Equation (7) by



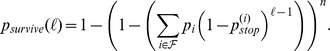



### Comparison of *Rcodon*, the Mixture Model and the EHEC Genome

The largest ORF in EHEC has a length of 5291 codons, which corresponds to a survival probability of ∼10-. The second largest ORF is 2793 codons long with a survival probability of ∼0.43. The survival probability can be interpreted as a p-value against the hypothesis that ORFs exceeding length 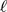 will be observed. The length limit with p-value 0.01 for an ORF in the model is 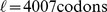 (Figure 2, right panel). In conclusion, the model cannot explain the single exceptionally large ORF in EHEC, but all others. This one ORF belongs to a rare class of giant genes (see [35] for more details). Similar results have been obtained for organisms of different GC-content (*Streptobacillus moniliformis*, NC_013515, GC-content 26.3% and *Xanthomonas campestris*, NC_007086, GC-content 65%; comparison in Supporting Information S1).

To further assess the functionality of the model, several global parameters of the mixture model prediction, *Rcodon* and EHEC were compared. First, the predicted total number of all ORFs in all six reading frames is 217461 in the mixture model, which is quite close to the 219368 ORFs observed in EHEC and 216184 in *Rcodon*. Second, the number of genes, or annotated ORFs, is 5901 in the mixture model, 5225 in EHEC, and 5827 in *Rcodon*. We are further interested in the 75% quantile of the ORF lengths. Seventy-five percent of all ORFs were predicted to be shorter than 35 codons in the mixture model, which is the same value for *Rcodon*. The EHEC genome has as similar, but somewhat lower, value of 33 codons. The deviation is due to a slight excess of longer ORFs. Further, the average ORF length is predicted to be 32:12 codons in the mixture model, 32:41 codons in *Rcodon* and 31:14 codons in EHEC. In summary, prediction, artificial genome and the natural EHEC genome show a close correspondence of global values.

### Comparison of *Rcodon*, the Mixture Model and Bacteria with Different GC-contents

We applied our mixture model to a total of 70 bacteria with GC-contents ranging from 21.4% to 74.9% (see Table S1). The individual models were assessed by several characteristics important for the global description of the genomes. For instance, expected average open reading frame lengths over different GC-contents, ORF frequencies and ORF length quantiles [2], [4], [5], [36] were compared between the predictions of the models and the bacterial genomes. Probability distributions were compared in a Quantile-Quantile-Plot (QQ-Plot, [37]).

**Figure 3:**
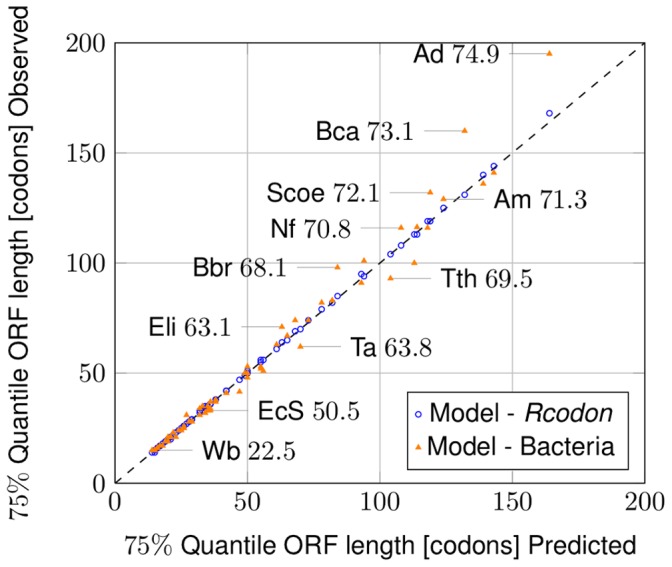
QQ-Plot. Comparison of 75% quantile of ORF lengths predicted by the mixture model to the ORF lengths observed in the natural genomes (orange triangles) and *Rcodon* (blue open dots), respectively. Some individual data points are labeled with an abbreviated species name and the corresponding GC-content according to Table S1.

**Figure 4:**
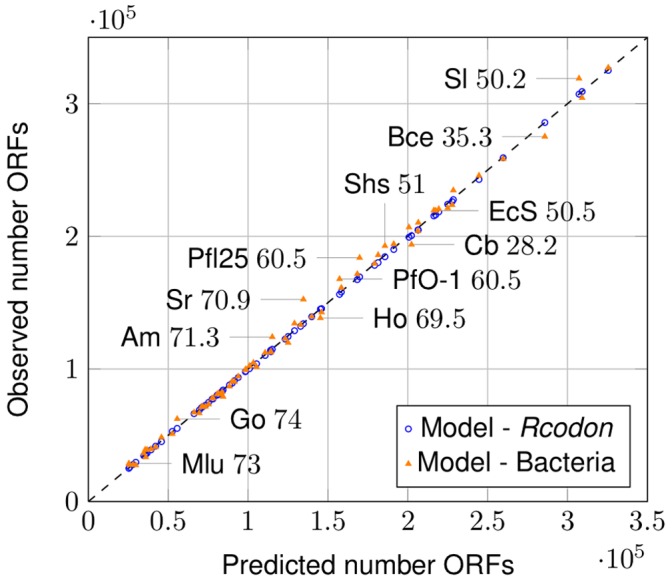
ORF number prediction. Comparison of ORF numbers predicted by the mixture model to the ORF numbers found in natural genomes (orange triangles) and *Rcodon* (blue open dots), respectively. Some individual data points are labeled with an abbreviated species name and the corresponding GC-content according to Table S1.

A quantile separates the given data into subsets. The 75% quantile of the ORF lengths is the boundary of length 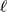 where 75% of all observed ORFs are shorter than 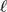. As can be seen from Figure 3 the values of *Rcodon* compared with the prediction of the model (blue open dots) form a straight line with slope =1, indicating not only correlation, but virtually identical distributions. Also, a clear correlation between the bacterial genomes and the model can be seen (Figure 3, orange triangles). When the number of ORFs predicted by the model was compared with the number of ORFs found in the bacterial genomes (Figure 4, orange triangles) or *Rcodon* (Figure 4, blue open dots), respectively, an excellent correlation was found.

**Figure 5:**
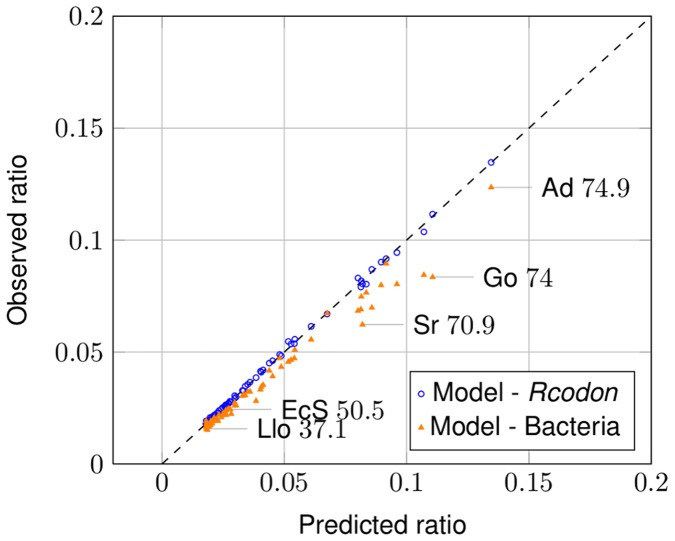
Ratio of annotated ORFs to non-annotated ORFs. The ratio predicted by the mixture model is compared to the ratio observed in bacterial genomes (orange triangles) and *Rcodon* (blue open dots), respectively. The observable slight difference between natural genomes and *Rcodon* is due the fact that the expected number of short coding ORFs in *Rcodon* deviates from the natural genomes (compare to Figure 7). Some individual data points are labeled with an abbreviated species name and the corresponding GC-content according to Table S1.

If the ratio of coding to non-coding ORFs is compared, *Rcodon* and the model are nearly identical (Figure 5, blue open dots). Interestingly, when comparing bacterial genomes with the model, most genomes show an excellent correlation, but genomes with increasing GC-content deviate to some extent (Figure 5, orange triangles).

**Figure 6:**
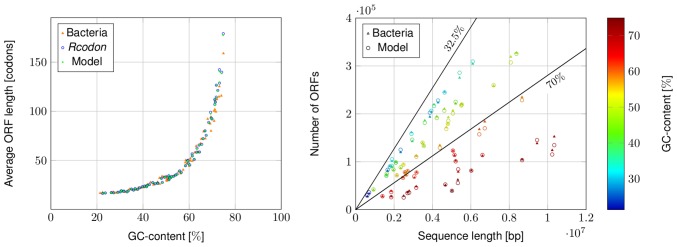
Influence of GC-content and sequence length. Left panel: Comparison of the average ORF lengths over the GC-content as predicted by the mixture model (green dots) compared to bacterial genomes (orange triangles) and *Rcodon* (blue open dots), respectively. Right panel: Comparison of the predicted number of ORFs to the observed number for different bacteria over sequence length. The number of ORFs expected depends on the sequence length and GC-content. The upper bounds for the number of ORFs expected are shown for the GC-contents 32.5% and 70%.

The effect of the GC-content on the model predictions was studied in more detail, as this is an important factor for the expected number of ORFs and the average ORF length [2], [38]. GC rich sequences have less stop codons, since the AT-rich stop codons *TAA,TAG,TGA* are increasingly rare [2]. Less stop codons cause longer ORFs (Figure 6, left panel). This correlation is reproduced in the mixture model, when calculating the average ORF length following Equation (4)
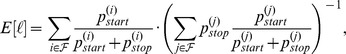
which depends only on the start and stop codon probabilities and is independent of the sequence length 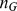, if 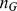 is sufficiently large.

The total length of a genome also influences ORF numbers since it determines the probability to observe very long ORFs and, trivially, a larger genome will harbor more ORFs. Therefore, the correlation of the number of ORFs found in all reading frames was compared with the genome length. When adding information about the GC-content, the pattern shown in Figure 6 (right panel) emerges. As said, longer genomes should in general contain more ORFs, but the actual number also depends on the GC-content, more precisely on the codon usage, as well. For example, if two organisms with roughly the same genome length of ∼6·10^6^bp, but different GC-contents of ∼71% and of ∼34% are compared, only ∼8·10^4^ ORFs for the high GC-content genome, but ∼3·10^5^ ORFs for the low GC-content genome were observed. This behavior is also reflected in the predictions of the model, showing only a minor variance in comparison with the natural genomes. The expected absolute number of ORFs in all reading frames,
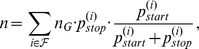
depends on the sequence length 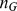 as well as the probabilities of start and stop codons, hence the GC-content (Figure 6, right panel). An upper bound for the number of ORFs observable in a genome over different sequence lengths is added to (Figure 6, right panel) at the example of a GC-content of 32.5% and for a relatively high GC-content of 70%. From a theoretical point of view, no organism with a GC-content of 70% can have more ORFs, than the bound labeled with 70% at a concrete sequence length. The derivation of this bound together with the reason for choosing GC-content 32.5% can be found in Supporting Information S1.

### Special Case Genus *Mycoplasma*

Even for *Mycoplasma mycoides* (NC_005364) with a GC-content of 24%, belonging to a very peculiar group of bacteria without a cell wall and a parasitic life style, the model is applicable. Of the three stop codons in the universal genetic code, only *TAA* and *TAG* are used in most mollicutes [39]. The model was adapted to this species which uses only two stop codons. The number of all ORFs matches well when the model was compared to *Mycoplasma mycoides* (60979 predicted versus 59911 observed). The predicted number of genes is 1106, which is close to the currently annotated number of 1017. The ORF distribution (Supporting Information S1), the 75% quantile of 17 codons and the average ORF length of 19:1 codons is reproduced by the model with 18:9 codons.

### Influence of the Coding ORF Lengths on the Prediction

**Figure 7:**
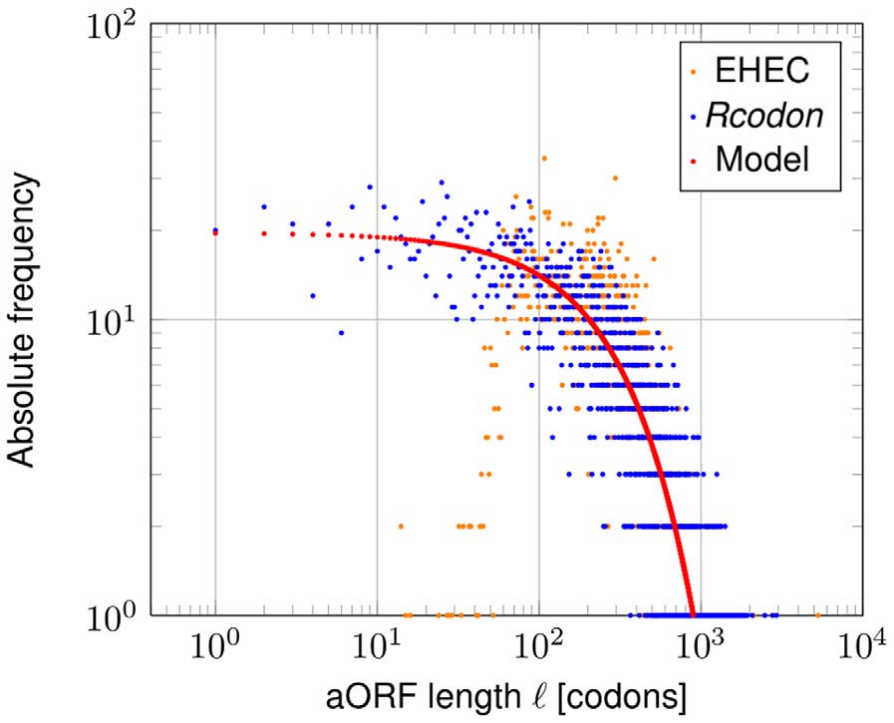
aORF lengths distributions. The absolute frequency of aORF lengths in codons from the EHEC genome (NC_002695) is compared to its *Rcodon* and the prediction of the mixture model. The visible difference between the natural genome and the theoretical expectations either by *Rcodon* or the mixture model is due to the fact that short ORFs are generally less likely to be annotated as functional proteins. However, this is changing (e.g., [40]) and short ORFs are picked up for annotations more frequently.

The model prediction, as well as *Rcodon*, shows a systematic error, as the number of coding ORFs (aORFs) in reading frame +1 is slightly over-estimated (Table 1). This can be also seen in Figure 5, in which all data points comparing the model with natural genomes (orange triangles) are slightly shifted to the right compared to the model and *Rcodon* (blue open dots). This deviation is due to the fact that the lengths of the annotated genes in bacteria do not follow a geometric distribution (Figure 7). The shortest aORF in EHEC has a length of 14 codons, but the model and *Rcodon* take all ORF lengths into account. Additionally, below ∼80 codons, less aORFs are annotated due to biological reasons (e.g., [12]). Consequently, the model expects more of those than are annotated. However, this is not considered as a problem. It is well known that current genome annotations rarely pick up short ORFs which results in an underestimation [40]. In Table 1, an excerpt of the predicted number of aORFs compared with the number of ORFs observed is presented. Using the arbitrary constraint of an individual minimal gene length for each organism, the predicted number of aORFs is even closer to the observed number (Table 1).

**Table 1:** Number of aORFs predicted and observed.

Accession	GC [%]	Number aORFs Natural	Shortest aORF 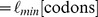	Number aORFsPredicted	Number aORFs 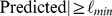
NC_011047	21.4	479	30	572	522
NC_005364	24	1017	36	1107	994
NC_009089	29.1	3693	21	4250	3989
NC_014251	39.8	2275	30	2475	2221
NC_005966	40.4	3306	23	3625	3383
NC_013730	50.2	6514	25	7264	6795
NC_002655	50.4	5266	12	5921	5709
NC_002695	50.5	5225	14	5901	5652
NC_006085	60	2297	33	2487	2259
NC_007492	60.5	5722	24	6243	5831
NC_007086	65	4271	30	4944	4540
NC_007509	65.3	1209	44	1376	1208
NC_006361	70.8	5683	34	6139	5536
NC_013595	70.9	8936	30	10198	9338
NC_013757	74	4801	30	5540	5084
NC_007760	74.9	4346	38	4663	4194

### Application of the Mixture Model to Not-annotated ORFs in Alternative Reading Frames

**Figure 8:**
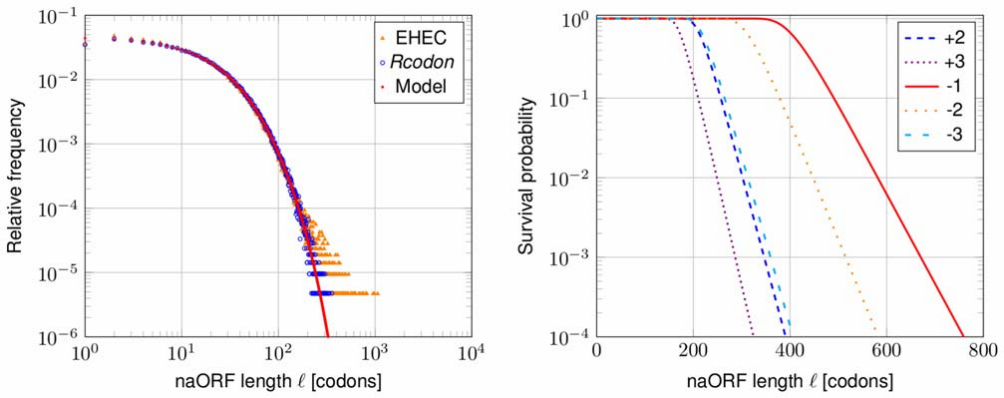
naORF lengths distributions. Left panel: The relative frequencies of naORF lengths derived from EHEC (orange triangle) are compared to *Rcodon* (open blue dots) and the mixture model (red line). Right panel: The survival probabilities of naORF lengths for the different alternative frames are derived from the mixture model. The survival probability shows the likelihood to observe at least one naORF with given length 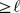. Indeed, longer naORFs are expected in reading frames −1 and, to some extent, frame −2 (see text).

**Figure 9:**
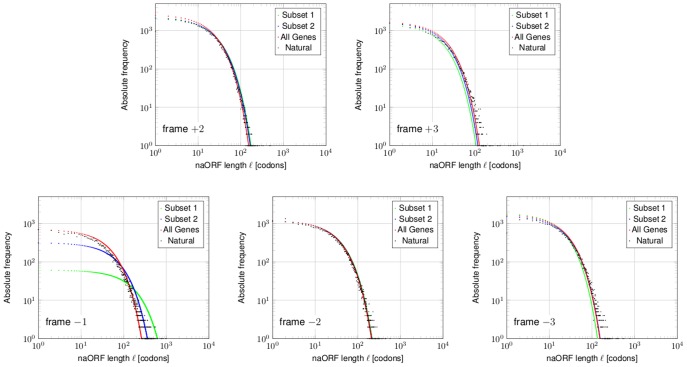
Length distributions of different groups of genes for each alternative frame. Shown are the absolute frequencies of naORF lengths for the genome of *E. coli* MC4100 (NC_000913) as predicted by the mixture model. Each colored line represents a different group used to obtain a codon usage as input to the model. Subset 1 of very high expressed genes is shown in green, Subset 2, contains, in addition to Subset 1, further highly expressed genes and is shown in blue (data from [41]). The group which includes all genes is shown in red. Finally, the natural frequencies obtained from the bacterial genome are shown in black triangles. In most alternative frames, the expression values of the annotated frame is of negligible influence, but not so for frame −1. As Silke [38] has already stated, most, but not all, long overlapping ORFs in −1 frame might be explained by a codon usage bias for highly expressed genes. However, this finding is not true for any other alternative frame nor for genes not highly expressed.

If, as hypothesized in the introduction, overlapping genes exist in bacteria, alternative open reading frames might contain non-annotated protein coding ORFs, which have been overlooked. How could these be detected? The mixture model distinguishes between “coding” ORFs in frame +1 and non-coding ORFs (naORFs) in any alternative frame. Therefore, the overall model that predicts overall ORF number statistics can easily be adapted to the non-coding case (Supporting Information S1). One would expect that the parameters for the naORFs in the model (based on valid statistical assumptions as shown above) will deviate from the parameters of the naORFs in the natural genomes. If a minimal initial stage of beneficial protein expression is established by random mutational events, selection will prevent decay back to the original state. Indeed, the naORF-length distribution of the model deviates significantly from the length distribution of the natural genome (Figure 8, left panel). Due to the different probabilities to obtain stop codons in the alternative frames, based on codon occurrences in the +1 reading frame, the survival probabilities differ for each frame (Figure 8, right panel). For instance, a long ORF of 600 codons observed in reading frame −1 is more probable than an ORF of the same size in reading frame +3. Taken this fact into account there is a number of long naORFs in each reading frame that cannot be explained statistically. Especially for reading frame −1 the observation of long ORFs overlapping coding sequences on the sense strand is not new, e.g., Merino *et al.* [14] described their findings as non-random phenomenon in 1994. The still unanswered question was: Have evolutionary forces shaped those long naORFs? Before this question can be answered, artifacts caused by biased codon usage (e.g., as known for highly expressed genes) have to be excluded first (e.g., [38]). Ishihama *et al.* [41] published a list of high and very high expressed genes of *E. coli* strain, MC4100 (NC_000913). We applied our mixture model to this organism and compared the predictions with the genome data for the individual alternative reading frames (Figure 9. Note that the absolute numbers of naORFs per frame are presented and are compared with the expected number of naORFs predicted by the model). Three groups of genes were defined and for each group the codon usage was used as input for the model. First, all annotated genes, next, Subset 1, which contains genes with the most highest expression and finally, Subset 2, which contains Subset 1 plus further highly expressed genes according to [41]. Comparing the group of all genes (Figure 9, red) to Subset 1 or Subset 2, a clear deviation occurs (Figure 9, green and blue, respectively), showing that indeed highly expressed genes can cause long ORFs in the −1 frame statistically as observed by Silke [38]. However, especially the codon usage of the very high expressed genes, which are about only 5% of all genes (Figure 9, green), causes this behaviour. But this is not true for any of the other alternative reading frames. Thus, long ORFs in +2, +3, −2 and −3 (Figure 9, black triangles) are not explained by any biased codon usage of the annotated ORF, which suggests that evolutionary forces have indeed shaped longer naORFs. It can be hypothesized that such long ORFs might be coding and form overlapping shadow genes. An interesting coincidence might be the fact that the recently described regulatory gene *adm* overlaps *dmdR1* in frame −2 in *Streptomyces* [25]. Concerning long overlapping ORFs in frame −1, it should be further noted that at least some of these ORFs appear in genes not found to be highly expressed [41]. Thus, long ORFs in frame −1 in lowly expressed genes are “significant” as well. Only the global length distribution of ORFs in frame −1 is indeed dominated by the biased codon usage of highly expressed genes.

### Concluding Remarks

This paper introduces a simple geometric mixture model that is able to reproduce statistical properties of bacterial genomes without empirical “curve fitting”. It depends on the actual codon usage of the organism examined and reproduces the overall number of ORFs, the overall length distribution and many other parameters of natural genomes. Most parameters between the model and the natural genome are in excellent agreement. As mentioned, all possible start codons were taken into account. While one could argue that *TGG* and *CTG* are rare codons and should be excluded from the simulations, all start codons are used in bacteria and can be changed by evolutionary processes to increase or decrease translational efficiency [29]. However, different choices of start codons were tested, both, the model prediction and the results of *Rcodon* fit the overall ORF length distribution of the corresponding organism (data not shown). Furthermore, it might be asked if the IID assumption is an oversimplification which influences the predictions of the model. Therefore, neighbouring biases among codons have been studied using a first order Markov chain to derive the stationary codon usage for reading frame +1. The predictions of both models – IID approach and first order Markov chain – are virtually identical (data not shown).

Statistical properties of ORFs are important in the context of shadow genes, a phenomenon generally accepted in viruses and bacteriophages [9], [42], but neglected in bacteria. The term “shadow gene” (borrowed from [10]) in this work refers to extensive overlaps in which two genes share the same DNA locus or are genes even embedded one in the other. Trivial overlaps (<30 codons) exist in basically all bacteria. Up to 30% of the genes of a bacterium may overlap trivially [13], but only few of the shadow genes have been recognized as true genes in bacteria to date (e.g., [21]–[25], [43], [44]). Using the model developed in this study, we could show that the length distribution of shadow genes overlapping annotated genes deviates significantly between model and bacterial genome. Thus, bacterial genomes contain a larger number of long shadow ORFs than expected based on statistical analysis. Random mutational drift would have eliminated the signal long ago, if no selection pressures were stabilizing shadow ORFs. Deviations between the statistical model and bacterial genomes directly call for a functional explanation, since selection is the only force known to stabilize the depletion of stop codons. Most shadow genes have escaped discovery, as they are dismissed as false positives in most genome annotation programs (e.g., [11]). This is in sharp contrast to many embedded overlapping genes that have been discovered in bacteriophages (e.g., [8], [42]). Since phages reside in a long term evolutionary equilibrium with the bacterial host genome [45], we suggest that overlooked shadow genes also exist in bacterial genomes [44]. Experimental verification of new protein coding sequences in prokaryotes is still a challenging task, as experiments are time consuming and expensive. In contrast to trivial approaches where just the largest ORFs are considered being candidates for protein coding genes, we could show that shorter than average ORFs also can be significant, depending in which alternative frame they appear. Furthermore, any observation indicating that genomes deviate from the model are a good starting point for further analysis since, most likely, biological specifics due to unknown functions may have shaped these differences during evolution.

All results presented in this paper apply to bacterial genomes only. Although intergenic regions appear in bacterial genomes, they represent a small fraction of the overall genome (for example 12.9% for EHEC), and, therefore, we decided to ignore them in our model. The eukaryotic genome organization, in contrast, is very different. It will be a non-trivial task to develop a more general model that can be applied to eukaryotic genomes also. Such a model needs to account for both, an exon and an intron sequence adjacent to one another at the same time. Whether a general model describing the length distributions of ORFs in both prokaryotes and eukaryotes can be designed at all is an open question.

## Materials and Methods

### Definition of ORFs, aORFs and naORFs

The ORF finder program takes the whole nucleotide sequence of an natural organism into account and finds all ORFs in all six reading frames. An ORF is defined as the longest string of triplets beginning with a start codon. An exception was made for annotated ORFs (aORFs), for which the annotated start codon was taken as beginning, ignoring any upstream start codons. The ORF finder was implemented in Python programming language and delivers the reading frame of the ORF, the first position of the start codon and the last position of the stop codon, the length of the ORF in base pairs and the length of the corresponding codons. Trivially, the not-annotated ORFs (naORFs) are all ORFs not annotated yet. The mapping of a naORF to a concrete reading frame is conducted in relation to the annotated genes by identifying overlaps with aORFs.

### Genome Sequence Data

We investigated a total of 70 genomes of fully sequenced bacteria reflecting different GC-contents ranging from 21.4% to 74.9% (see Table S1). The complete genome data was downloaded as GenBank entry from NCBI database via Entrez. From those files we extracted the whole genome sequence as well as the annotated gene positions. Some genome sequences contain undefined nucleotides. Those positions were substituted with concrete nucleotides as given in Table 2 (similar to [4]). Additionally, all annotated genes were ignored if their length was no multiple of three or contained undetermined positions in the region of the start or stop codon.

**Table 2:** Undetermined nucleotides and their substitutions [47].

Symbols	Possible substitutions	Origin and designation
R	A,G	puRine
Y	C,T	pYrimidine
M	A,C	aMino
K	G,T	Ketone
S	C,G	Strong interaction
W	A,T	Weak interaction
B	C,G,T	not-A, B follows A in alphabet
D	A,G,T	not-C, D follows C in alphabet
H	A,C,T	not-G, H follows G in alphabet
V	A,C,G	not-T/U, V follows U in alphabet
N	A,G,C,T	aNy

### Derivation of the Model

Our model results from an approximation of the random genome *Rcodon*. Basically, it is derived by assuming independence of the different reading frames, as well as using certain average case analyses. For an introduction to the fundamental concepts of probability theory used in this section see, e.g., Feller [46].

#### Reading frames +1 and −1

In the reading frames +1 and −1 the sequence generated by the model consists of 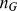 independent and identical distributed (IID) random codons 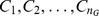, where 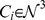, 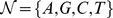 is drawn according to the codon usage 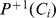 of the original genome, based on the annotated genes. The codon usage is the number of occurrences of each codon in a string of all concatenated annotated ORFs divided by the number of all codons in that string. The length 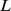 of an ORF in an individual reading frame 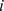 follows a geometric distribution
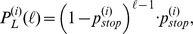
where 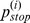 denotes the stop codon probability in the corresponding frame. For 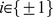 the stop codon probability is determined by the codon usage of the natural genome taking the sum of the three probabilities for the stop codons. The geometric distribution is used in general modeling waiting time of a process and was already applied in the context of ORF lengths by [2].

#### Reading frames +2, +3, −2 and −3

In the reading frames +2, +3, −2 and −3 the situation is slightly different compared to frames 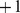 and 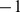. The sequence 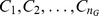 is not IID anymore, but is a Markov chain with memory one for each frame 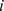. The transition probabilities from codon 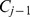 to codon 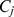 in frame 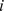, denoted with 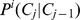, are induced by the distribution of the codons in the 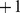 frame. For example, in the frame 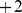 the transition probabilities can be obtained according to the following approach: Each codon 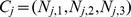 consists of nucleotides 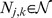, resulting in
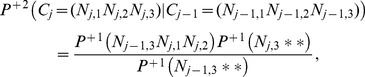
(2)where * denotes the sum over all probabilities for each possible nucleotide combination. An example that illustrates the derivation of Equation (2) can be found in Supporting Information S1.

**Figure 10:**
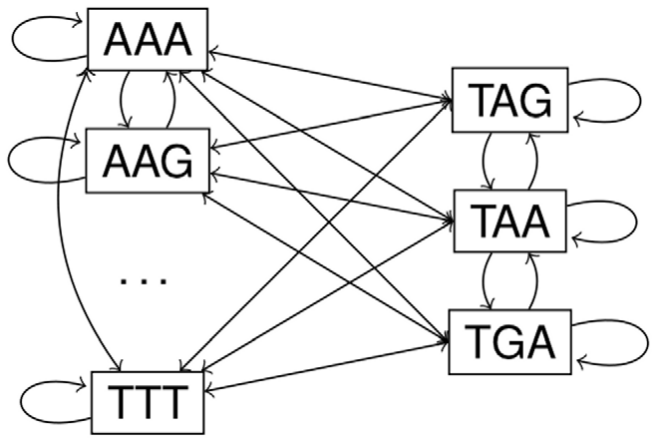
Ergodic Markov chain. Markov Chain connects all codons. For each reading frame the stationary of this ergodic Markov chain is calculated to obtain the individual start and stop codon probabilities.

The Markov chains (Figure 10) for the different frames are ergodic (hence aperiodic and irreducible) if all codon probabilities in the +1 frame are positive, as this implies that all transition probabilities between the codons are positive (see Equation (2)). The stop codon probabilities for each reading frame 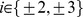 can then be obtained from the stationary distribution of the corresponding Markov chain.

#### Mixture model

The probability to observe exactly one ORF of length 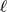 in any of the six reading frames 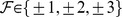 can be calculated by a weighted sum over all six geometric distributions
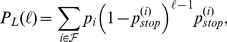
(3)where 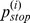 is the stop codon probability in reading frame 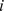 and 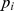 is determined by the distribution of the ORFs in the different reading frames.

**Figure 11:**
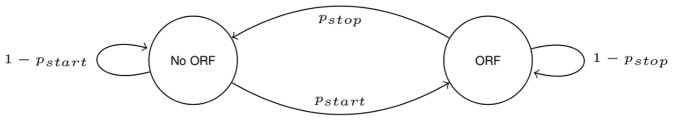
Two state Markov chain. Stationary distribution of this Markov model reveals the probability for being within an ORF.

To calculate the parameter 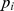, we have to consider the number of ORFs in each reading frame. This can be conducted using an average case analysis. First, observe that in a certain frame 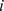 a fraction 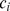 of the whole sequence is within an ORF, whereas a fraction 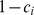 is between two consecutive ORFs. The 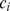 are obtained from the stationary distribution of the two state Markov chain (Figure 11). The evaluation of the stationary distribution leads to
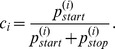



For each frame 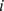 the length of the genome sequence 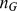 in codons consists of a coding part of length
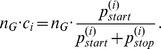



The expected ORF length, observed within such a coding region is the expected value of the geometric distribution
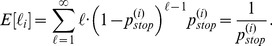
(4)


Applying this knowledge, we are now interested in the number of ORFs in reading frame 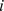 denoted as 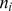 using the following equality
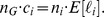



Therefore, the number of ORFs in reading frame 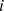 can be calculated by
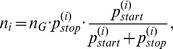
(5)whereby the parameter 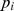 for an individual reading frame 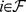 is given by
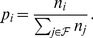
(6)


#### Survival Limit of the Model

From the probability to observe exactly one ORF of length 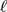 in Equation (3), we can derive the probability to observe an ORF of length 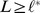


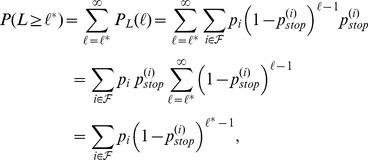
where the last step follows from the geometric series.

The probability to observe 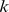 ORFs with length 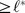 in 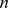 trials follows a Binomial distribution
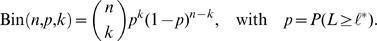



The survival probability was defined as the probability to observe at least one ORF with length 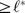


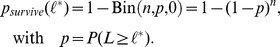
(7)


All probabilities can be compared to the relative frequencies of ORF lengths in the natural organisms. If the absolute number of ORFs with an probability *p* is needed, the expected number of ORFs in *n* trials is calculated by
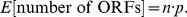



## Supporting Information

Table S1 **Bacterial species investigated in this study. Species names, accession numbers, GC-content and length of the analyzed organism.**
(PDF)

Supporting Information S1 **Additional Data and Figures.** This file contains the detailed derivation of the upper bound on the number of ORFs observable (Section 1). An example how the transition probabilities of the Markov chain are calculated (Section 2). Length distributions and survival probabilities of further organisms (Section 3). Comparison of naORF parameters in bacteria (Section 4).
(PDF)
