# Supplementary material for: Predicting Statistical Properties of Open Reading Frames in Bacterial Genomes
Source: PLoS One. 2012 Sep 24;7(9):e45103. doi: 10.1371/journal.pone.0045103 (PMC3454372; doi:10.1371/journal.pone.0045103)
Supplement: Table S1 — Bacterial species investigated in this study. Species names, accession numbers, GC-content and length of the analyzed organism. (PDF) [file pone.0045103.s001.pdf]

Species	Abbr.	GC [%]	Length [bp]	Accession
Candidatus *Phytoplasma mali*	CPm	21.4	601943	NC_011047
Wigglesworthia brevipalpis	Wb	22.5	697724	NC_004344
Streptobacillus moniliformis DSM 12112	Strm	26.3	1662578	NC_013515
Arcobacter butzleri RM4018	Ab	27	2341251	NC_009850
Clostridium botulinum A str. ATCC 19397	Cb	28.2	3863450	NC_009697
Clostridium difficile 630	Cdif	29.1	4290252	NC_009089
Campylobacter jejuni RM1221	Cj	30.3	1777831	NC_003912
Clostridium acetobutylicum ATCC824	Ca	30.9	3940880	NC_003030
Clostridium ljungdahlii DSM 13528	Clj	31.1	4630065	NC_014328
Francisella tularensis subsp. *holarctica* FTNF002-00	Fth	32.2	1890909	NC_009749
Staphylococcus aureus subsp. *aureus* JH1	Staa	33	2906507	NC_009632
Flavobacterium johnsoniae UW101	Fj	34.1	6096872	NC_009441
Bacillus cereus ATCC 14579	Bce	35.3	5411809	NC_004722
Anaerococcus prevotii DSM 20548	Ap	36.1	1883067	NC_013171
Legionella longbeachae NSW150	Llo	37.1	4077332	NC_013861
Listeria monocytogenes EGDe	Lm	38	2944528	NC_003210
Helicobacter pylori HPAG1	Hp	39.1	1596366	NC_008086
Streptococcus pneumoniae TCH8431/19A	Strp	39.8	2088772	NC_014251
Acinetobacter sp. ADP1	Asp	40.4	3598621	NC_005966
Streptococcus suis 05ZYH33	Strs	41.1	2096309	NC_009442
Gardnerella vaginalis 409-05	Gv	42	1617545	NC_013721
Bacteroides fragilis NCTC 9343	Bf	43.2	5205140	NC_003228
Campylobacter curvus 525.92	Cc	44.5	1971264	NC_009715
Xenorhabdus bovienii SS-2004	Xb	45	4225498	NC_013892
Tropheryma whipplei TW08/27	Tw	46.3	925938	NC_004551
Haliscomenobacter hydrossis DSM 1100	Hh	47.1	8371686	NC_015510
Yersinia enterocolitica subsp. *enterocolitica* 8081	Yee	47.3	4615899	NC_008800
Porphyromonas gingivalis W83	Pg	48.3	2343476	NC_002950
Geobacillus thermodenitrificans NG80-2	Gth	49	3550319	NC_009328
Spirosoma linguale DSM 74	Sl	50.2	8078757	NC_013730
Nitrosococcus oceani ATCC 19707	No	50.3	3481691	NC_007484
Paenibacillus sp. JDR-2	Ps2	50.3	7184930	NC_012914
Escherichia coli O157:H7 EDL933	EcE	50.4	5528445	NC_002655
Escherichia coli O157:H7 Sakai	EcS	50.5	5498450	NC_002695
Pectobacterium wasabiae WPP163	Pw	50.5	5063892	NC_013421
Shigella sonnei Ss046	Shs	51	4825265	NC_007384
Salmonella enterica subsp. *enterica* serovar Agona str. SL483	See	52.1	4798660	NC_011149
Corynebacterium diphtheriae NCTC 13129	Cdiph	53.5	2488635	NC_002935
Magnetococcus sp. MC-1	Msp	54.2	4719581	NC_008576
Mobiluncus curtisii ATCC 43063	Mc	55.4	2146480	NC_014246
Renibacterium salmoninarum ATCC 33209	Rsa	56.3	3155250	NC_010168
Cronobacter turicensis z3032	Ct	57.4	4384526	NC_013282
Paenibacillus mucilaginosus KNP414	Pm	58.4	8663821	NC_015690
Bifidobacterium dentium Bd1	Bd	58.5	2636367	NC_013714
Granulibacter bethesdensis CGDNIH1	Gbe	59.1	2708355	NC_008343
Propionibacterium acnes KPA171202	Pac	60	2560265	NC_006085
Pseudomonas fluorescens PfO-1	PfO-1	60.5	6438405	NC_007492

Continued on next page

Table S1 – continued from previous page

Species	Abbr.	GC [%]	Length [bp]	Accession
Pseudomonas fluorescens SBW25	Pfl25	60.5	6722539	NC_012660
Corynebacterium jeikeium K411	CjK	61.4	2462499	NC_007164
Laribacter hongkongensis HLHK9	Lh	62.4	3169329	NC_012559
Erythrobacter litoralis HTCC2594	Eli	63.1	3052398	NC_007722
Thermanaerovibrio acidaminovorans DSM 6589	Ta	63.8	1848474	NC_013522
Mycobacterium abscessus ATCC 19977T	Ma	64.1	5067172	NC_010397
Xanthomonas campestris pathovar *campestris*	Xcc	65	5079002	NC_007086
Burkholderia sp. 383 3	Bsp	65.3	1395069	NC_007509
Pseudomonas aeruginosa LESB58	Pae	66.3	6601757	NC_011770
Gordonia bronchialis DSM 43247	Gbr	67.1	5208602	NC_013441
Bordetella bronchiseptica RB50	Bbr	68.1	5339179	NC_002927
Thermus thermophilus HB8	Tth	69.5	1849742	NC_006461
Haliangium ochraceum DSM 14365	Ho	69.5	9446314	NC_013440
Nocardia farcinica IFM10152	Nf	70.8	6021225	NC_006361
Streptosporangium roseum DSM 43021	Sr	70.9	10341314	NC_013595
Kocuria rhizophila DC2201	Kr	71.2	2697540	NC_010617
Amycolatopsis mediterranei U32	Am	71.3	10236715	NC_014318
Kytococcus sedentarius DSM 20547	Ks	71.6	2785024	NC_013169
Streptomyces coelicolor A3(2)	Scoe	72.1	8667507	NC_003888
Micrococcus luteus NCTC 2665	Mlu	73	2501097	NC_012803
Beutenbergia cavernae DSM 12333	Bca	73.1	4669183	NC_012669
Geodermatophilus obscurus DSM 43160	Go	74	5322497	NC_013757
Anaeromyxobacter dehalogenans 2CP-C	Ad	74.9	5013479	NC_007760
