# Supplementary material for: Predicting Statistical Properties of Open Reading Frames in Bacterial Genomes
Source: PLoS One. 2012 Sep 24;7(9):e45103. doi: 10.1371/journal.pone.0045103 (PMC3454372; doi:10.1371/journal.pone.0045103)
Supplement: Supporting Information S1 — Additional Data and Figures. This file contains the detailed derivation of the upper bound on the number of ORFs observable (Section 1). An example how the transition probabilities of the Markov chain are calculated (Section 2). Length distributions and survival probabilities of further organisms (Section 3). Comparison of naORF parameters in bacteria (Section 4). (PDF) [file pone.0045103.s002.pdf]

SUPPORTING INFORMATION:

Predicting Statistical Properties of Open Reading Frames in Bacterial Genomes

Katharina Mir[1,*], Klaus Neuhaus[2], Siegfried Scherer[2], Martin Bossert[1], Steffen Schober[1]
1 Institut für Nachrichtentechnik, Universität Ulm, Albert-Einstein-Allee 43, 89081 Ulm, Germany
2 Lehrstuhl für Mikrobielle Ökologie, Department für Grundlagen der Biowissenschaften am WZW, Technische Universität München, Weihenstephaner Berg 3, 85350 Freising, Germany
∗ E-mail: katharina.mir@uni-ulm.de

1 Derivation of upper bound on number of ORFs observable

The shortest ORF lengths are observed under the assumption that all nucleotides in the genome are independent and identically distributed, matching a given GC-content. Therefore, an upper bound can be obtained for the expected number of ORFs in this "worst case" scenario. This bound reflects the maximal number of ORFs, that can be observed in a given genome. Under the assumption of IID nucleotides, the probability for an ORF is independent of the reading frame, hence all reading frames are equally likely. Following the notation of [2], the frequencies of the nucleotides are denoted by $f_C = f_G = p$ and $f_A = f_T = q$. The stop codon probability is the sum over all three stop codon frequencies $p_{stop} = f_T f_A^2 + 2 f_T f_A f_G = q^2 - q^3$ [2]. The same principle was used to obtain the start codon probability

$$p_{start} = f_A f_T f_G + f_T f_T f_G + f_C f_T f_G + f_G f_T f_G = \frac{1}{2} q - q^2.$$

Clearly $q = \frac{1-(f_G+f_C)}{2}$ depends only on the GC-content considered. Therefore, the upper bound on the number of ORFs depends on the GC-content as well as the sequence length and is given by

$$n \leq 6 \cdot n_G \cdot p_{stop} \cdot \frac{p_{start}}{p_{stop} + p_{start}} = 2 \cdot n_G^{[\text{bp}]} \cdot \left(q^2 - q^3\right) \cdot \frac{\frac{1}{2} q - q^2}{\frac{1}{2} q - q^3}, \tag{1}$$

where $n_G^{[\text{bp}]}$ denotes the sequence length in base pairs. The relative number of ORFs, independent of the sequence length, is the fraction $n_{rel} = 2 \cdot p_{stop} \cdot \frac{p_{start}}{p_{stop}+p_{start}}$, which depends only on the GC-content via q and yields the maximal value at a GC-content of $\sim 32.5\%$ in Figure S1 (left). This bound was calculated over different sequence lengths for a GC-content of 32.5%, which is the maximum value of Equation (1) and for a relatively high GC-content of 70% (Figure 6, right panel). From a theoretical point of view, no organism with a GC-content of 70% can have more ORFs, than the bound labeled with 70% at a concrete sequence length. The line with GC-content 32.5% is the overall upper bound.

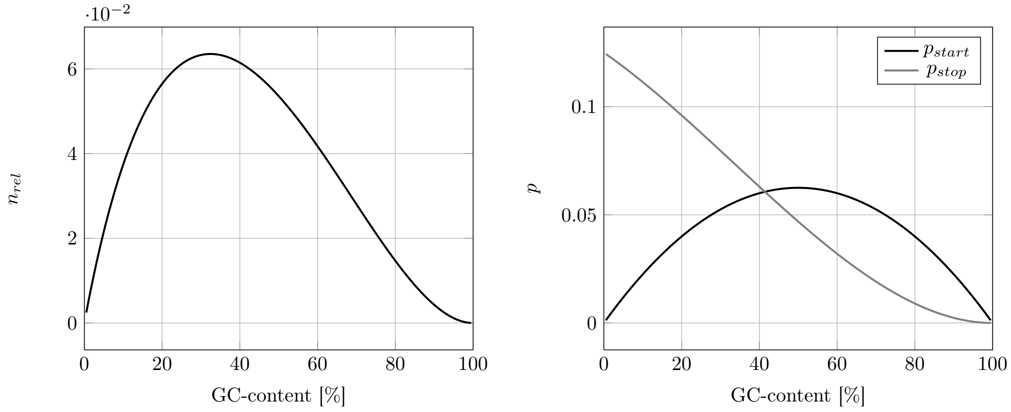

Figure S1. Relative number of ORFs over GC-content. Left Panel: The upper bound on the relative number of observable ORFs depending on the GC-content is shown. Note that the bound is independent of the sequence length. To obtain the maximal number of ORFs for an IID nucleotide model with six independent reading frames, the value has to be multiplied with the sequence length of the organism in base pairs. Right Panel: The corresponding start and stop codon probabilities, respectively, over the GC-content are shown. Note that bacterial organisms have GC-contents in the range of 21.4% to 74.9%.

2 Example to calculate transition probabilities

An example of how the pattern of nucleotides is denoted in the reading frame +1 based on reading frame +2 is shown in Figure S2. Each nucleotide $N_{j,k} \in \mathcal{N}$ belongs to a concrete codon $C_j = (N_{j,1}, N_{j,2}, N_{j,3})$. N_* denotes arbitrary nucleotides. If we consider reading frame +2, the transition probability $P^{+2}(C_2 \mid C_1)$ depends on the codon probability in reading frame +1. Precisely

$$\begin{aligned} P^{+2}\left(C_2 = (N_{2,1}N_{2,2}N_{2,3}) \mid C_1 = (N_{1,1}N_{1,2}N_{1,3})\right) &= \frac{P^{+1}(*N_{1,1}N_{1,2})P^{+1}(N_{1,3}N_{2,1}N_{2,2})P^{+1}(N_{2,3}**)}{P^{+1}(*N_{1,1}N_{1,2})P(N_{1,3}**)} \\ &= \frac{P^{+1}\left(N_{1,3}N_{2,1}N_{2,2}\right)P^{+1}\left(N_{2,3}**\right)}{P^{+1}\left(N_{1,3}**\right)}, \end{aligned}$$

where $*$ denotes the sum over all probabilities for each possible nucleotide combination. A similar calculation can be realized for the other reading frames −2, +3 and −3 as well.

$$\begin{array}{r||ccccccccc} +1 & N_* & N_{1,1} & N_{1,2} \mid & N_{1,3} & N_{2,1} & N_{2,2} \mid & N_{2,3} & N_* & N_* \\ +2 & N_* \mid & N_{1,1} & N_{1,2} & N_{1,3} \mid & N_{2,1} & N_{2,2} & N_{2,3} \mid & N_* & N_* \end{array}$$

Figure S2. Reading frames feedback to +1. Excerpt for reading frames +1 and +2.

3 Further Organisms

Figures S3, S4, S5, S6, S7, and S8 show three more organisms as examples of the length distributions and survival probabilities. Figures S3 and S4 show the length distributions and survival probabilities of *Mycoplasma mycoides* subsp. *mycoides* SC with GC-content 24%, genome length 1211703 bp and accession number NC_005364. Figures S5 and S6 show the length distributions and survival probabilities of *Streptobacillus moniliformis* DSM 12112 with GC-content 26.3%, genome length 1662578 bp and accession number NC_013515. Figures S7 and S8 show the length distributions and survival probabilities of *Xanthomonas campestris* pathovar *campestris* with GC-content 65%, genome length 5079002 bp and accession number NC_007086.

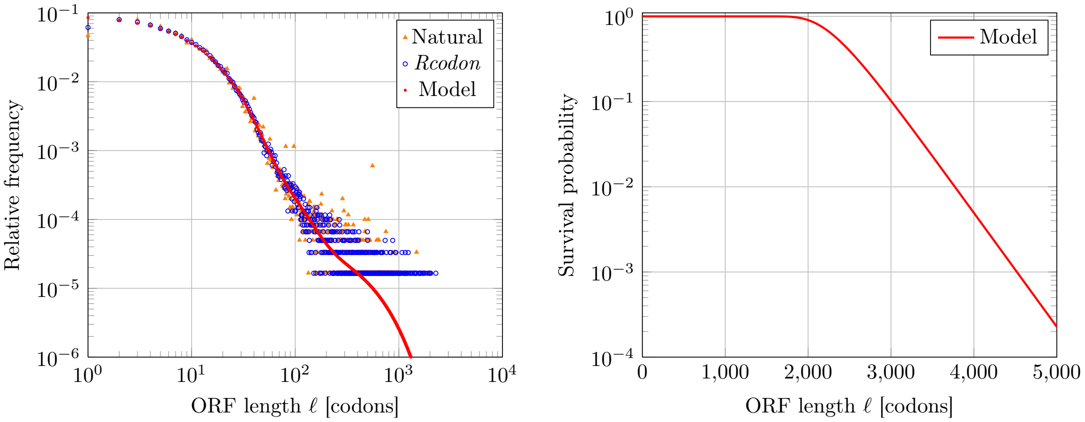

Figure S3. ORF lengths distribution and survival probability of *Mycoplasma*. Left panel: Shown is the relative frequency of ORF lengths in codons of *Mycoplasma mycoides* subsp. *mycoides* SC (NC_005364). This bacterium has a GC-content of 24% and uses only two stop codons (TAA and TAG). Compared are the natural ORF lengths (orange triangles) with *Rcodon* (blue open dots) and the prediction of the mixture model (red). Right panel: The survival probability (probability to observe at least one ORF with given length $\geq \ell$ in any of the six reading frames) is derived from the mixture model.

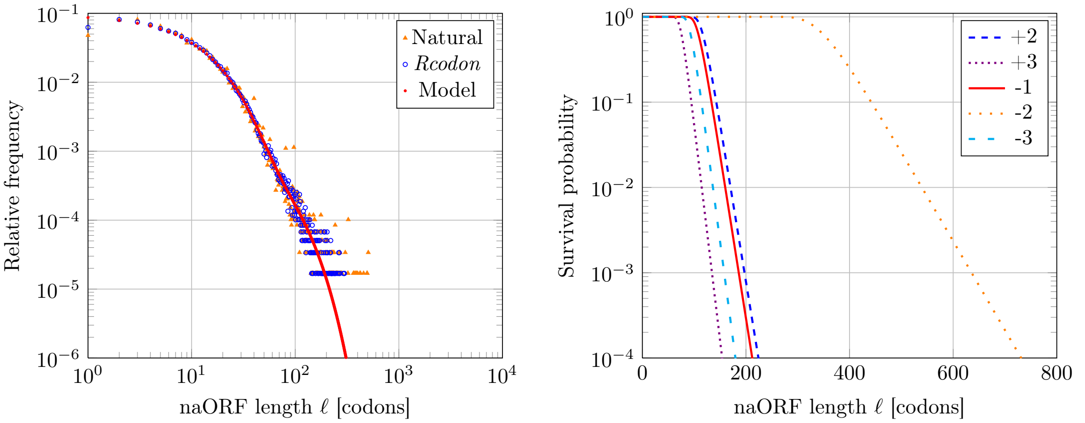

Figure S4. naORF lengths distributions and survival probability of *Mycoplasma*. Left panel: Shown is the relative frequency of naORF lengths in codons of *Mycoplasma mycoides* subsp. *mycoides* SC (NC_005364). This bacterium has a GC-content of 24% and uses only two stop codons (TAA and TAG). Compared are the natural naORF lengths (orange triangles) with *Rcodon* (blue open dots) and the prediction of the mixture model (red). Right panel: The survival probabilities of naORF lengths for the different alternative frames are derived from the mixture model. The survival probability shows the likelihood to observe at least one naORF with given length $\geq \ell$. Indeed, longer naORFs are expected in reading frames -2 than, e.g., in reading frame $+2$.

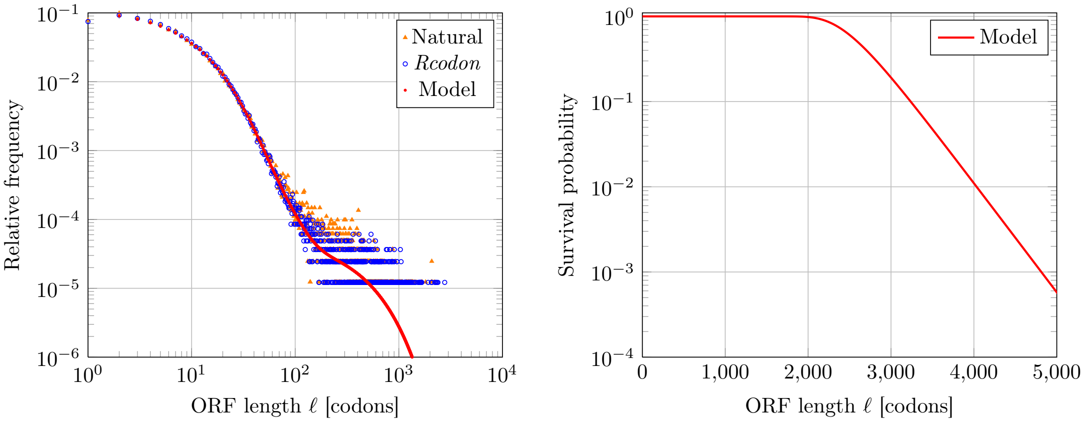

Figure S5. ORF lengths distribution and survival probability of *Streptobacillus*. Left panel: Shown is the relative frequency of ORF lengths in codons of *Streptobacillus moniliformis* (NC_013515). This bacterium has a GC-content of 26.3%. Compared are the natural ORF lengths (orange triangles) with *Rcodon* (blue open dots) and the prediction of the mixture model (red). Right panel: The survival probability (probability to observe at least one ORF with given length $\geq \ell$ in any of the six reading frames) is derived from the mixture model.

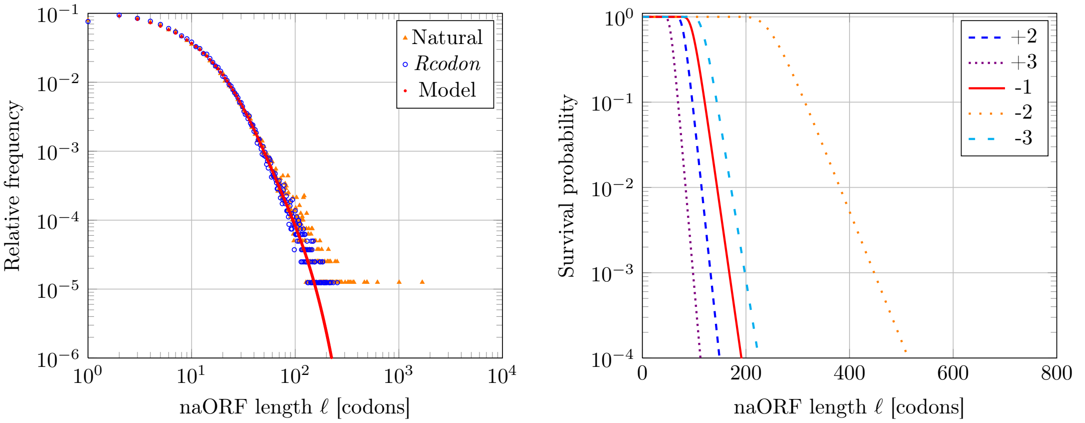

Figure S6. naORF lengths distributions and survival probability of *Streptobacillus*. Left panel: Shown is the relative frequency of naORF lengths in codons of *Streptobacillus moniliformis* (NC_013515). This bacterium has a GC-content of 26.3%. Compared are the natural naORF lengths (orange triangles) with *Rcodon* (blue open dots) and the prediction of the mixture model (red). Right panel: The survival probabilities of naORF lengths for the different alternative frames are derived from the mixture model. The survival probability shows the likelihood to observe at least one naORF with given length $\geq \ell$. Indeed, longer naORFs are expected in reading frames -2 than, e.g., in reading frame -3.

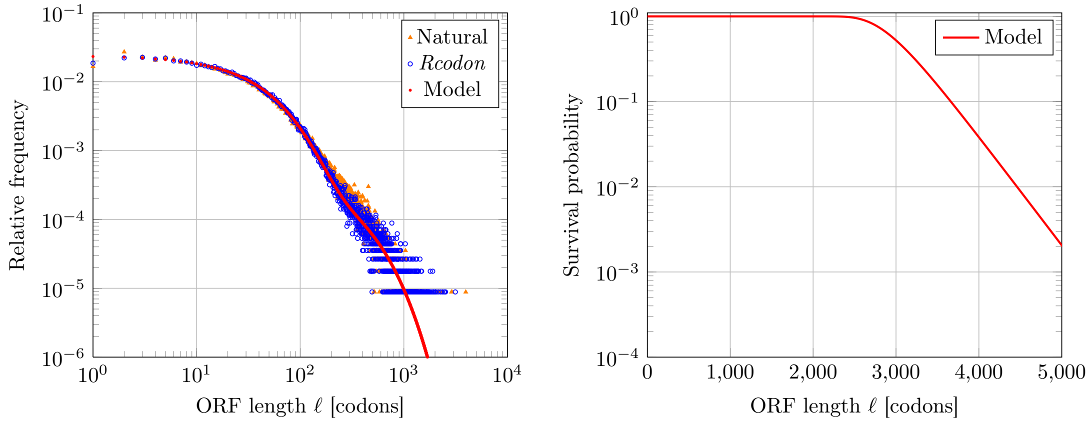

Figure S7. ORF lengths distribution and survival probability of *Xanthomonas*. Left panel: Shown is the relative frequency of ORF lengths in codons of *Xanthomonas campestris* (NC_007086). This bacterium has a GC-content of 65%. Compared are the natural ORF lengths (orange triangles) with *Rcodon* (blue open dots) and the prediction of the mixture model (red). Right panel: The survival probability (probability to observe at least one ORF with given length $\geq \ell$ in any of the six reading frames) is derived from the mixture model.

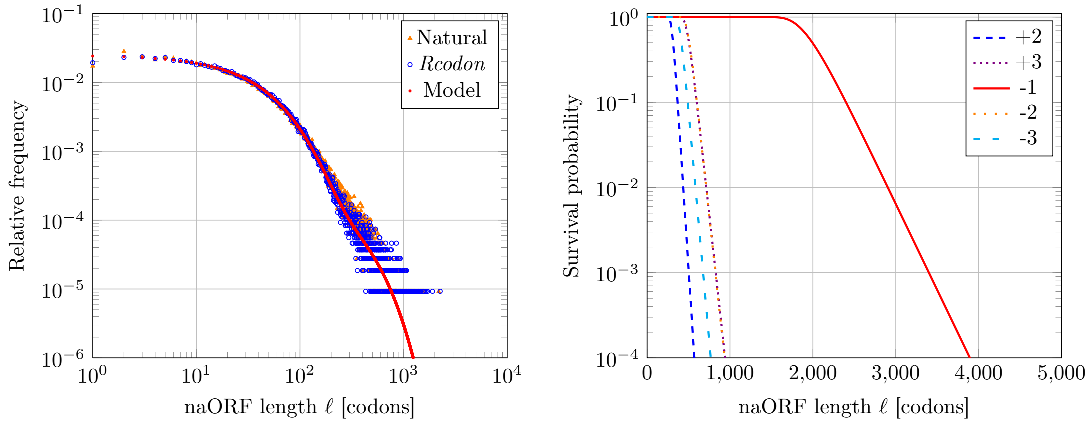

Figure S8. naORF lengths distributions and survival probability of *Xanthomonas*. Left panel: Shown is the relative frequency of naORF lengths in codons of *Xanthomonas campestris* (NC_007086). This bacterium has a GC-content of 65%. Compared are the natural naORF lengths (orange triangles) with *Rcodon* (blue open dots) and the prediction of the mixture model (red). Right panel: The survival probabilities of naORF lengths for the different alternative frames are derived from the mixture model. The survival probability shows the likelihood to observe at least one naORF with given length $\geq \ell$. Indeed, longer naORFs are expected in reading frames -1 than, e.g., in reading frame -2.

4 Comparison of naORF parameters in Bacteria

Figure S9 shows the 75% quantile of the naORFs. The correlation of *Rcodon* compared to the prediction of the model (blue open dots) as well as between the natural genomes and the model (orange triangles) can be seen. The same observation holds, if we compare the number of naORFs predicted by the model with the number of naORFs found in the natural genomes (Figure S10, orange triangles) or *Rcodon* (Figure S10, blue open dots), respectively.

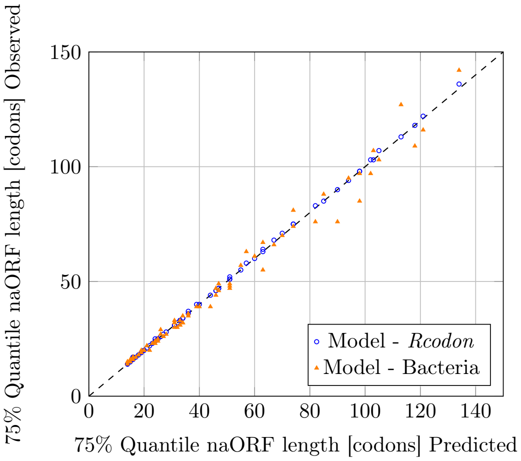

Figure S9. QQ-Plot of naORF lengths for different bacteria. Comparison of 75% quantile of naORF lengths predicted by the mixture model to the naORF lengths observed in the natural genomes (orange triangles) and *Rcodon* (blue open dots), respectively. The plot shows a clear correlation between the predictions of the mixture model and the 70 bacteria of different GC-content investigated (Table S1.

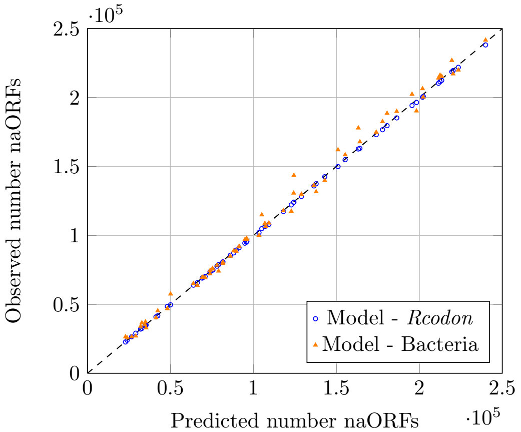

Figure S10. naORF number prediction for different bacteria. Comparison of naORF numbers predicted by the mixture model to the naORF numbers found in natural genomes (orange triangles) and *Rcodon* (blue open dots), respectively. The plot shows a clear correlation between the predictions of the mixture model and the 70 bacteria of different GC-content investigated (Table S1).
